# Combined with Bone Marrow-Derived Cells and rhBMP-2 for Osteonecrosis after Femoral Neck Fractures in Children and Adolescents: A case series

**DOI:** 10.1038/srep30730

**Published:** 2016-08-01

**Authors:** Fuqiang Gao, Wei Sun, Wanshou Guo, Bailiang Wang, Liming Cheng, Zirong Li

**Affiliations:** 1 Centre for Osteonecrosis and Joint-Preserving & Reconstruction, Department of Orthopedics, Beijing Key Laboratory of Arthritic and Rheumatic Diseases, China-Japan Friendship Hospital, National Health and Family Planning Commission of the People’s Republic of China, Beijing 100029, China

## Abstract

Osteonecrosis of the femoral head (ONFH) following femoral neck fractures is a rare, yet severe, disorder in children and adolescents. This study evaluated the effectiveness of core decompression (CD) combined with implantation of bone marrow–derived cells (BMDC) and rhBMP-2 for osteonecrosis of femoral head (ONFH) after femoral neck fractures in children and adolescents. This study included 51 patients, aged 11.4–18.1 years, with ARCO stages I–III ONFH after femoral neck fractures between 2004 and 2010. The hips were divided into two groups based on whether the lateral pillar of the femoral head (LPFH) was preserved: LPFH and non-LPFH groups. All patients were followed up clinically and radiographically for a minimum of 5 years. 44 patients (86.3%) had improved clinical outcome. Radiologically, 9 of the 51 hips (17.6%) exhibited collapse onset or progression of the femoral head or narrowing of the hip joint space, and one patient in the non-LPFH group required hip arthroplasty due to the worsened syndrome. The technique provided an effective therapeutic option for children and adolescents with ONFH following femoral neck fractures. It relieves hip pain and prevents the progression of osteonecrosis in young patients lasting more than 5 years after surgery.

Osteonecrosis of the femoral head (ONFH) following femoral neck fractures is a rare, yet severe, disorder in children and adolescents^1^,^2^. The development of osteonecrosis has been demonstrated to directly correlate with the severe long-term consequences such as femoral head collapse, and its progression usually leads to impaired hip joint function in early stages of the disease^2^,^3^. The blood supply to the femoral epiphysis transits away from the metaphysis during infancy and evolves toward the lateral epiphyseal vessels during childhood before finally recovering its ultimate blood supply from an alliance of vessels^1^,^2^,^3^. This transition causes the epiphysis to be particularly vulnerable to damage after femoral neck fracture during childhood and adolescence. ONFH has been shown to occur in up to 40% of hips after femoral neck fractures^4^. Regardless of the treatment, the prevalence of osteonecrosis remains high in children and adolescents^2^, and to date no clear guidelines exist for successfully treating osteonecrosis in the pediatric population.

However, compared with adults, adolescents with osteonecrosis may have their own slightly favorable characteristics^5^. They have thick articular cartilage, which may be more resistant to deformation and disruption of the femoral head^5^. Besides, adolescents may also tolerate some subsidence of the necrotic fragment without disruption of the articular surface^5^,^6^. The treatment principle of childhood ONFH should be tailored to handle the differences with the osteonecrosis of adults. Reconstructing support to the joint surface before cartilage fracture is becoming increasingly vital to preserve joint function^5^,^6^,^7^. Core decompression (CD) is one of the methods most frequently used for the treatment of early-stage ONFH, which could relieve the intraosseous pressure improving venous return and promoting the vascularization of the necrotic area of the femoral head^8^. However, it cannot facilitate osteoanagenesis in the necrotic area^8^,^9^,^10^,^11^,^12^,^13^. It has been proved that bone marrow–derived cells (BMDC) containing the bone mesenchymal stem cells (BMSCs) provide osteoblasts and enhance bone formation^8^,^14^. Relatively recent studies have demonstrated that the rhBMP-2 might improve the clinical efficacy and quality of bone repair^15^. Core decompression procedures can be further optimized for treating ONFH. Therefore, now it is the right time to reinvent the way to better treat juvenile osteonecrosis.

It is believed that core decompression combined with implantation of BMDC and rhBMP-2 may improve clinical outcomes for osteonecrosis after femoral neck fractures in this age group. Therefore, the goal of this retrospective study was to evaluate the effectiveness of core decompression combined with implantation of autologous BMDC, which contained a high concentration of BMSCs, and rhBMP-2 for osteonecrosis after femoral neck fractures in children and adolescents based on the clinical and radiographic results in a case series.

## Results

**Figure 1:**
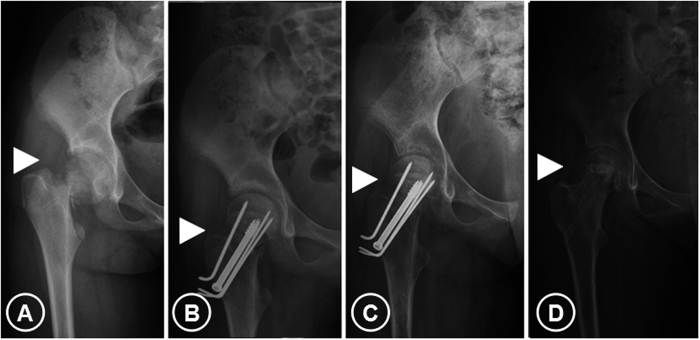
The first patient, a 13-year-old girl: one case of osteonecrosis after femoral neck fracture. (**A**) X-ray showing displaced femoral neck fracture (Delbet Type II). (**B**) X-ray image after internal fixation. (**C**) X-ray image 15 months after internal fixation showing good healing of femoral neck fracture segments after surgery; then she underwent the surgery for internal fixation removal. (**D**) X-ray image 2 months after internal fixation removal when she felt pain in her right hip showing the necrotic lesion of right femoral head and sclerosis zone formation around the necrotic bone (arrowheads).

**Figure 2:**
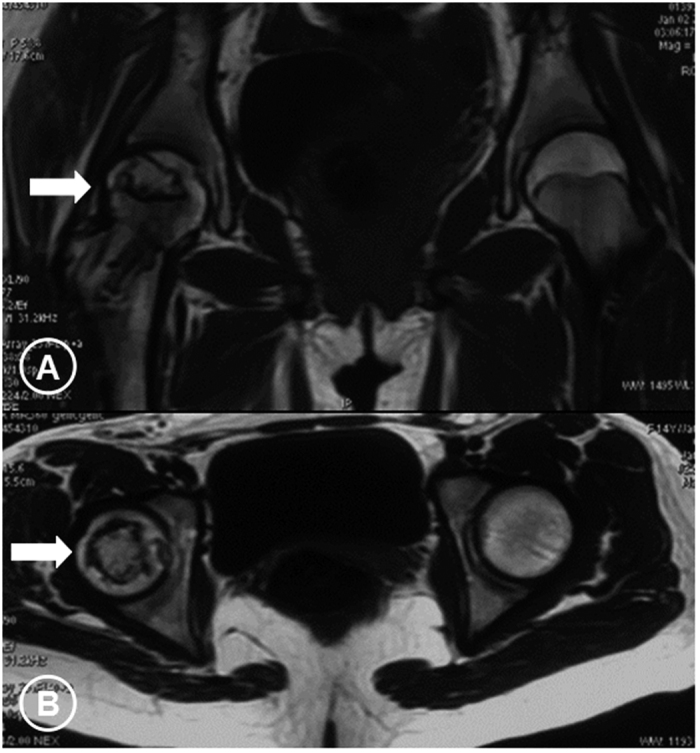
The coronal and axial MRI scan images of the same patient mentioned in Fig. 1 showing the necrotic lesion of the right hip (ARCO Stage IIc, CJFH Type L1) (arrows).

Between January 2004 and September 2010, 56 patients (56 hips) with early ONFH following femoral neck fractures in children and adolescents were recruited in the institution. This study included patients with Stages I–III ONFH according to the Association Research Circulation Osseous (ARCO) classification^16^. Patients with Stage IV, acute infection, malignancies, coagulation disorders, myelodysplastic syndrome, and anemia were excluded. Five patients were dropped from the study due to incomplete information or loss to follow-up. The details of 51 patients included in this study are shown in Table 1. They comprised 38 males and 13 females with a mean age of 16.3 years (range, 11.4–18.1 years) at the time of surgery. The femoral neck fractures classified according to Delbet classification^17^ included type I in 5 patients (5 hips), type II in 31 patients (31 hips), and type III in 15 patients (15 hips). Of 51 cases with femoral neck fractures in children, 2 cases were treated by external fixation with plaster spica of hip and 49 cases were treated by internal fixation with K-wires and (or) cannulated screw combined with capsular decompression plus spica. Forty-nine cases (96%) had displaced fractures and only two cases (4%) had nondisplaced fractures. Among these, 31 cases occurred after internal fixation removal and 18 cases retained the whole internal fixation. The time interval from internal fixation removal to diagnosis of osteonecrosis was 5.9 ± 3.8 months. The underlying diagnosis was confirmed by anteroposterior (AP) and frog-leg lateral radiographs or magnetic resonance imaging (MRI) scans (Figs 1 and 2). All cases received an MRI evaluation according to China-Japan Friendship Hospital (CJFH) classification^18^,^19^ for ONFH based on three pillars. The hips were divided into two groups based on whether the lateral pillar of the femoral head (LPFH) was preserved^20^ (Table 2). The LPFH group consisted of 23 hips with the preservation of LPFH (including CJFH types M, C, and L1). The non-LPFH group consisted of 28 hips without the preservation of the LPFH (including CJFH types L2 and L3).

### Clinical outcome

In this study, the treatment was well tolerated and none of the severe complications were seen during or after the operation. The total duration of follow-up was 6.8 ± 1.5 years. A dramatic improvement was noted in pain scores and HHS after the treatment (Table 3). Clinically, 44 patients had an improved outcome; the overall success rate was 86.3%, and the clinical success rate was higher in the LPFH group than in the non-LPFH group (91.3% vs 82.1%). However, the differences were not statistically significant (*P* = 0.436). The Harris hip score and radiographic results indicated obvious postoperative improvement in all the cases. The Harris hip scores significantly increased from 71.7 ± 8.1 to 85.3 ± 5.5 (*P* < 0.001). The clinical success rate for hips with ARCO stages I+II was 95.8%; stage IIIA, 83.3%; and stage IIIB+IIIC, 66.7% (Table 3).

Table 4 summarizes the results of clinical assessment before and after the treatment in LPFH and non-LPFH groups. The mean VAS score for both the groups decreased from 4.6 ± 1.8 to 1.6 ± 0.8 in the LPFH group and from 6.1 ± 1.7 to 2.1 ± 0.9 (*P* < 0.001) in the non-LPFH group. The mean HHS for both the groups increased from 77.4 ± 5.6 to 87.5 ± 5.2 in the LPFH group and from 67.0 ± 6.7 to 83.5 ± 5.1 (*P* < 0.001) in the non-LPFH group. The results indicated that the improvement was mainly due to the reduction in pain. The patients in the non-LPFH group described higher VAS values and lower HHS values than those in the LPFH group (*P* < 0.01). Most patients in both the groups stated the daily life function as significantly improved, but the difference in the Harris score following the removal of the pain score after surgery in both the groups was statistically significant (*P* = 0.008). This might be associated with a more rapid progress of osteonecrosis without the support of lateral pillar in the non-LPFH group. At the last follow-up, CD surgery might have the potential to curtail the progression of the disease and delay the need for THA. However, for some cases with CJFH types L2 and L3 osteonecrosis, the treatment outcomes were poor or inadequate. One hip in the non-LPFH group, but none in the LPFH group, failed following femoral head collapse and required hip arthroplasty.

### Radiological outcome

**Figure 3:**
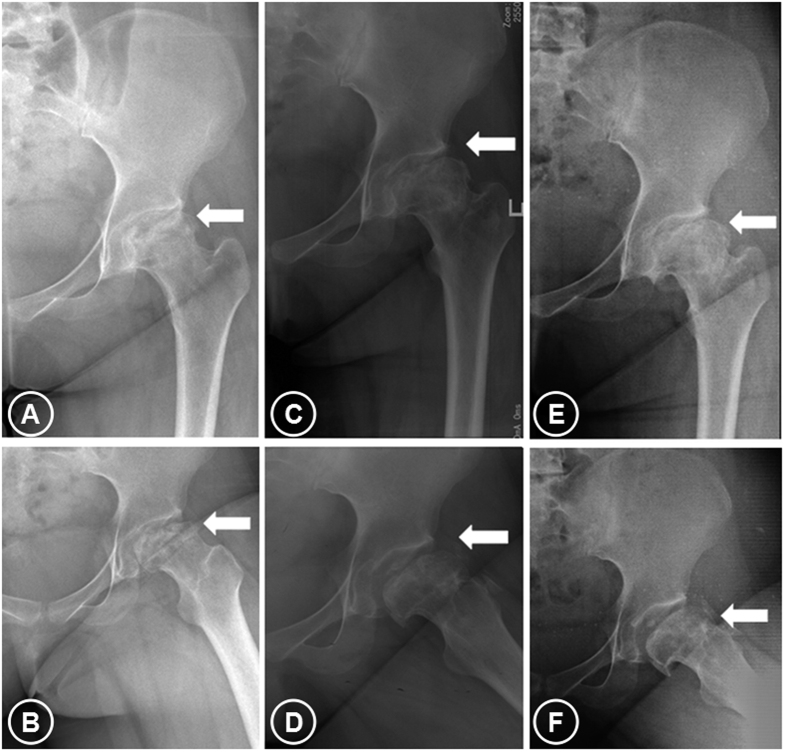
The second patient, 15-year-old girl: the osteonecrosis of the femoral head in her left hip 6 months after internal fixation removal following femoral neck fracture (ARCO Stage IIIc, CJFH Type L3). Preoperative x-ray (**A**, anteroposterior view; **B**, frog lateral view) showing already collapse of the necrotic lesion of the left femoral head after fracture; x-ray at 3 years after the combined treatment (**C**, anteroposterior view; **D**, frog lateral view), showing femoral head flattening and no further collapse, yet with stable reshaping; x-ray after 6 years (**E**, anteroposterior view; **F**, frog lateral view), showing no further collapse and the shape of the femoral head conserved (arrows).

Radiologically, 9 of the 51 hips (17.6%) exhibited collapse onset or progression of the femoral head or narrowing of the hip joint space, and 1 patient (2%) required hip arthroplasty due to the worsened syndrome. The overall radiographic success rate was 82.4% (Fig. 3), and the radiographic success rate was higher in the LPFH group than in the non-LPFH group (4.3% vs 28.6%). Meanwhile, the differences were statistically significant (*P* = 0.028). The screws in three cases were removed during surgery due to the extrusion and loosening of the screws seen on the x-ray films. The outcomes were not affected by the retention of the internal fixation devices of the femoral head. Radiographic failure was found in 8 (23.5%) of 34 cases without internal fixation (Fig. 3) and 1 (6.7%) of 15 cases with internal fixation. However, the differences were not statistically significant (*P* = 0.242). Multiple small drilling techniques across the growth plate did not result in growth retardation in these cases. Also, the epiphyseal growth plate in several cases appeared to be closed early after femoral neck fracture.

## Discussion

Osteonecrosis is one of the most serious complications after femoral neck fractures in children and adolescents and also is very difficult to deal with^1^,^2^,^3^,^4^. The special metaphyseal blood vessels make the femoral head in this age group more vulnerable to injury^21^. Children and adolescents are prone to osteonecrosis after femoral neck fractures because of the slender and variational blood supply to the femoral epiphysis^2^,^4^. Obviously a transition occurs between the pediatric and adolescent femoral epiphyseal blood supply, but this needs to be further investigated^2^. It has been suggested that osteonecrosis develops on account of direct damage to the vessel during the traumatic event, kinking of the vessels with displacement, intracapsular tamponade by hematoma, and injury during treatment or initiation of the clotting cascade within the lateral epiphyseal arteries^22^. So far no effective means of restoring blood supply to the femoral head with femoral neck fractures are available. ONFH occurs within 24 months following injury such as femoral neck fracture^1^,^2^,^22^,^23^,^24^,^25^,^26^. The time to diagnosis of ONFH in this study varied significantly. The time to osteonecrosis was 16.4 ± 9.2 months. Yeranosian *et al*.^27^ reported the overall rate of osteonecrosis following fracture across 30 studies as 23%. However, no consensus exists as to which of the risk factors are the most predictive of osteonecrosis after femoral neck fracture in children and adolescents. One meta-analysis^28^ provided statistical evidence that fracture type and age were identified as the only significant predictors of ONFH. Older children were more likely to develop ONFH after fracture. Fracture type according to Delbet classification and fracture displacement seem to be the most predictive of the development of osteonecrosis^2^. It was also found that most of the osteonecrosis (96%) occurred following displaced fractures. Perhaps displacement can directly injure or kink the dominating vessels supplying the femoral head.

Regardless of the treatment, the prevalence of osteonecrosis remains high in children and adolescents^1^,^2^, and to date no clear guidelines exist for successfully treating osteonecrosis in the pediatric population. The pediatric age group has a thick articular cartilage, which may be more resistant to deformation and disruption of the femoral head^5^. Besides, adolescents may also tolerate some subsidence of the necrotic fragment without disruption of the articular surface^5^,^6^. In spite of collapse onset or mild progression of the femoral head in some pediatric patients, reconstructing support to the joint surface earlier can prevent further collapse of the femoral head or postpone the emergence of osteoarthritis due to the thick cartilage safeguard so as to preserve joint function^6^,^7^. The adolescent age group with open growth plates may have a more favorable prognosis when treated later in the course of the disease^5^. Based on this, stage III ONFH with already collapse of the femoral head was included in this study. The purpose of core decompression was to remove necrotic bone, reduce venous congestion, and encourage revascularization^13^,^29^,^30^. It might reduce intraosseous pressure to decrease venous congestion and improve microvessel blood flow^31^. Vascularity of the proximal femur can be also enhanced by angiogenesis caused by the trephine opening new vascular channels^5^. In children, the growth plate of the proximal femur may become a barrier to revascularization^32^. So, the multidrilling of the epiphyseal plate might promote reossification of the femoral head^32^. Thus, core decompression is beneficial for revascularization of the femoral head. However, it cannot facilitate osteoanagenesis in the necrotic area^8^,^9^,^10^,^11^,^12^,^13^. A previous study showed that CD and autologous transplantation of bone marrow rich in BMSCs were quite effective and safe in treating adult ONFH^8^. It was proved that bone marrow–derived cells (BMDC) containing plenty of BMSCs provide osteoblasts and enhance bone formation^8^,^33^,^34^. Bone morphogenetic proteins were shown to not only enhance osteoinductive repair, but also stimulate new blood vessel formation^35^. Animal experiments concluded that sustained controlled release of rhBMP-2 above a therapeutic dose could penetrate the necrotic bone graft earlier, induce early callus wrapping of the necrotic bone, and promote angiogenesis and osteogenesis, thereby substituting inside of the dead bone^36^,^37^. Relatively recent studies demonstrated that rhBMP-2 might improve the clinical efficacy and quality of bone repair^15^,^38^. Therefore, it is believed that core decompression combined with implantation of autologous BMDC and rhBMP-2 was a valuable attempt for osteonecrosis after femoral neck fractures in children and adolescents.

The overall success rate was 86.3% (radiographic success 82.4%) in this study, and the clinical success rate was higher in the LPFH group than in the non-LPFH group (91.3% vs 82.1%). However, the differences were not statistically significant (*P* = 0.436). The clinical success rates of 95.8%, 83.3%, and 66.7% were obtained for stages I+II, stage IIIA, and stage IIIB+IIIC patients, respectively. The procedure achieved great success for early-stage ONFH (precollapse) and preferable clinical success in late-stage patients in spite of the femoral head collapse (postcollapse). The results showed the overall radiographic success rate to be 82.4%. Radiologically, nine hips (17.6%) exhibited collapse onset or progression of the femoral head or narrowing of the hip joint space, and one patient required hip arthroplasty due to the worsened syndrome. The radiographic success rate was higher in the LPFH group than in the non-LPFH group (*P* = 0.028). It is presumed that the collapse rate may be low and the femoral head maintains the spherical shape for a long time when the lateral pillar is preserved. Previous studies showed that whether ONFH progressed to collapse is determined by preservation of the lateral pillar^23^. This study confirmed that the necrotic foci, the lateral pillar of the femoral head, and the degree of involvement directly affect the treatment effect of surgery, especially for those with radiological appearance. Furthermore, in the case of femoral head flattening, yet with stable reshaping, children or adolescents can have no or mild pain and favorable hip function due to the suitable thick cartilage, so as to postpone the time of hip arthroplasty. It was found that the radiographic failure rate was also slightly higher in the patients without internal fixation compared with the patients with internal fixation. This might be because internal fixation support was likely to delay the collapse of femoral head. It is best not to remove the internal fixation before the adequate osteogenic repair of necrotic femoral head.

Improved clinical outcomes have been recorded following core decompression in the early stages of necrosis. A firm structural support in the decompressed area during the revascularization process helps to prevent disruption of the articular cartilage^5^,^39^. Gradual substitution of the necrotic bone or implant material by living bone reduces the decline of the bone’s mechanical properties to a minimum^40^. Several graft materials and adjunctive techniques have been applied following core decompression^6^,^8^,^12^,^13^,^29^,^30^,^34^,^35^,^36^,^37^,^38^. Hernigou *et al*.^33^ pioneered the clinical application of a cell-based strategy for the treatment of ONFH and reported on the postoperative outcome in 189 cases (116 patients) treated with the injection of autologous bone marrow concentrate^33^. Over the course of the 5- to 10-year follow-up, 9 out of 145 patients with early-stage ONFH and 25 out of 45 patients with later-stage ONFH required total hip replacement. Recently, one prospective, double-blind trial by Gangji *et al*.^34^ on the 5-year follow-up of 19 cases (24 hips) who received either CD alone or CD plus BMDC because of early-stage ONFH reported that 8 out of 11 hips in the CD group and only 3 out of 13 hips in the CD plus BMDC group revealed a disease progression with the structural disintegration of the subchondral bone. It has been already proved that implanted BMSCs promote both osteogenesis and angiogenesis in the femoral head^14^,^41^. These profitable effects may be mediated, at least to some extent, by BMSCs and endothelial precursor cells^41^. The retrospective clinical study over a 4-year follow-up by Calori *et al*.^12^ reported a total of 38 patients (40 hips) with early-stage adult ONFH treated using the CD technique combined with a biologically based approach such as BMSCs plus BMP with a flexible xenograft bone substitute. They found a significant reduction in both pain and joint symptoms and the incidence of fractures. At 36 months, 33 patients achieved clinical and radiographic healing. They confirmed that the combined CD technique may be an effective treatment for patients with early-stage osteonecrosis of the femoral head^12^. Tang *et al*.^42^ investigated the influence of BMSCs, transduced with either BMP-2 or β-galactosidase and seeded onto β-TCP scaffolds, on ONFH consolidation. Although control animals (core decompression only) showed ONFH progression with structural disintegration and collapse of the subchondral bone 4 months after treatment, no signs of such progression were observed in the cell-treated groups^42^. In the case of BMP-2 transduction, significantly higher amounts of new bone and higher maximum compressive strength and bone density were found^42^. Along with BMPs, surgeons have considered combining CD with BMSCs, which represent osteoprogenitor cells with the power to strengthen bone repair^12^. It has emerged as one of efficient approaches to enhance bone formation for ONFH.

There are several limitations associated with this study. This study is limited by virtue of the retrospective analysis. Because osteonecrosis after femoral neck fractures is relatively uncommon in children and adolescents, it would have been difficult to mount a randomized controlled trial. The sample size of this study was relatively small. Due to the limited number of cases, it is believed that the cell transplantation plus BMP-2 enhanced the success rate of CD compared with other researches, which should be further confirmed by future high-quality multicenter case-control studies. Considering the impact of the radiation, the CT scan was not used in regular follow-up, which may influence the radiological observation of the state of osteogenesis. Despite these limitations, however, it was considered that this retrospective study might be valuable to clarify a hypothesis, provided an available method, and focus on the study question.

In conclusion, core decompression combined with implantation of bone marrow–derived cells and rhBMP-2 can relieve hip pain and improve hip function in early osteonecrosis after femoral neck fractures in children and adolescents lasting more than 5 years after surgery. It might have the potential to curtail the progression of this disease and delay the need for THA. It may be the treatment of choice particularly in early-stage osteonecrosis of the femoral head. However, for some osteonecrosis without lateral pillars, the treatment outcomes were inadequate. The radiographic failure rate was also slightly higher in the patients without internal fixation compared with the patients with internal fixation. This might be because internal fixation support was likely to delay the collapse of femoral head. Furthermore, in the case of femoral head flattening, yet with stable reshaping, the children or adolescents can have no or mild pain, and favorable hip function due to suitable thick cartilage. In sum, this combined technique provided beneficial effects for hips affected by early- to middle-stage osteonecrosis after femoral neck fractures in the pediatric population in the relatively long term. Further prospective multicenter studies are needed to indicate a therapeutic potential of the combined technique for osteonecrosis after femoral neck fracture in the special age group.

## Methods

The study was approved by the Institutional Review Board on Human Studies of the Ethical Committee of our hospital, and the study procedures adhered to the 1975 Declaration of Helsinki. Informed consent was obtained from all the patients.

### Surgical procedures

#### Preparation of BMDC

**Figure 4:**
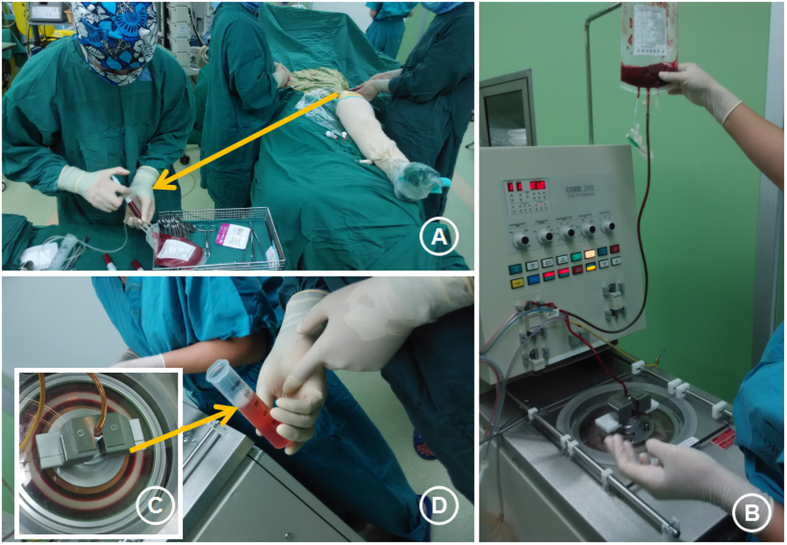
Bone marrow aspirate was harvested using sterile techniques from the anterior iliac crest (**A**) of the patient in the supine position under general or spinal anesthesia. A total of 100–200 mL of bone marrow (**A**) was aspirated. The harvested bone marrow was processed directly in the operating room using a cell concentrator–separator device (**B**, COBE 2991 Cell Processor, Terumo BCT, Gambro BCT, Inc., CO, USA) at 3000 rpm for 5–10 min (**B**) to allow for the separation of the bone marrow components. The bone marrow components were stratified according to the density of the plasma (**C**). Bone marrow mononuclear cells (BMMCs) and red blood cells were located from the inner layer to the outer layer (**D**).

Preoperative intravenous antibiotics were administered before surgery. Bone marrow aspirate was harvested using sterile techniques from the anterior iliac crest of the patient in the supine position under general or spinal anesthesia (Fig. 4). Bone marrow blood was collected using a trocar from the bilateral anterior superior iliac crest. The trocar was 6–8 cm in length and 1.5 mm in inner diameter. The trocar perforated the lateral cortical bone of the iliac bone and entered the cancellous bone. The trocar was pushed into the iliac bone in different directions in a fan-shaped manner. The bone marrow blood was transferred into an aseptic blood bag (containing anticoagulant natrium, sodium citrate, and glucose). A total of 50–60 mL of blood was collected at each needle site. Perforations were performed every 4–5 mm, and 100–180 mL of bone marrow blood was collected eventually.

The bone marrow blood was separated using an automatic blood cell processor (COBE 2991TM Cell Processor GAMBRO BCT. Inc.) at 2500 rpm for 5–10 min. The bone marrow components were stratified according to the density of the plasma. BMDC with bone marrow mononuclear cells (BMMCs) and red blood cells were located from the inner layer to the outer layer, while the BMSCs were gradually concentrated in the BMMC layers during centrifugation. This centrifugation reduced 100–180 mL of bone marrow to a concentrated suspension of approximately 30–50 mL of BMMCs (see Fig. 4).

#### Core decompression and implantation of BMDC

**Figure 5:**
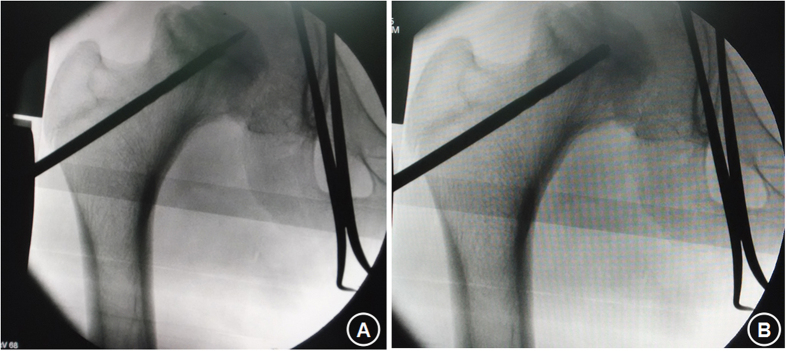
Intraoperative image showing core decompression treatment of ONFH. The decompression was done with a percutaneous approach using a 1.0-mm diameter Kirschner needle under X-ray guidance. Fluoroscopy-guided positioning of the pilot Kirschner wire into the necrotic lesion was performed in the AP and lateral plain view radiographs. Usually multiple small-diameter drill holes were made. Guided by the Kirschner wire, the hollow trephine was gently passed through the sclerotic zone of the necrotic lesion under fluoroscopic control in the anteroposterior (**A**) and lateral (**B**) plain view radiographs. The trephine should be 2–3 mm away from the cartilage. Debridement of the necrotic lesion was done through the drill hole.

**Figure 6:**
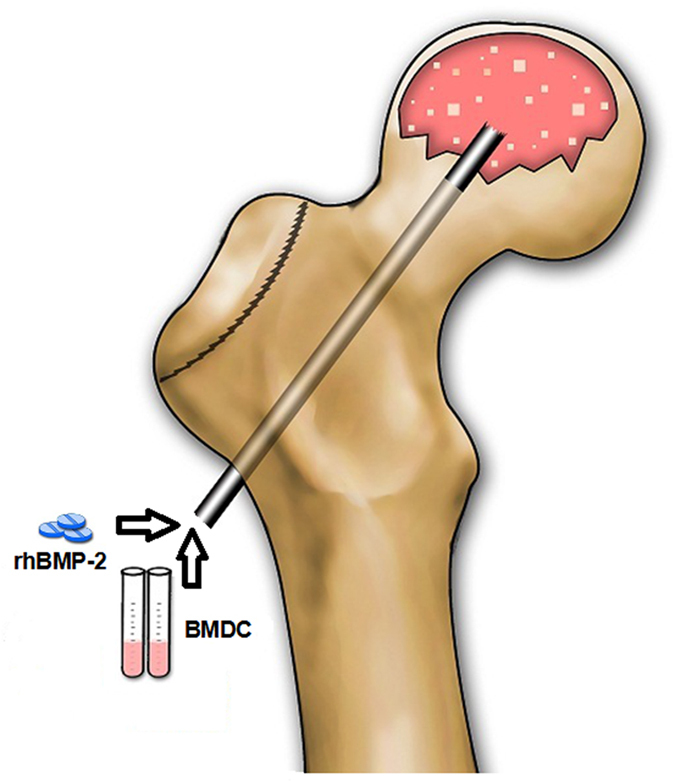
Schematic diagram of ‘the combined core decompression procedure’. The trephine should be 2–3 mm away from the cartilage. Usually multiple small-diameter drill holes were made. Then, the bone marrow–derived cells (BMDC) along with rhBMP-2 were injected into the femoral head using a small trephine with an inner diameter of 1.5 mm and an outer diameter of 3.5 mm. Bone wax was used to block the outlet of the channel and prevent leakage.

The operation was then conducted with the patient in the supine position. The decompression was done with a percutaneous approach using a 1.0-mm diameter Kirschner needle under X-ray guidance. Fluoroscopy-guided positioning of the pilot Kirschner wire into the necrotic lesion was performed in the anteroposterior (AP) and lateral plain view radiographs. Usually multiple small-diameter drill holes were made. Guided by the Kirschner wire, the hollow trephine was gently passed through the sclerotic zone of the necrotic lesion under fluoroscopic control in the anteroposterior (A) and lateral (B) plain view radiographs. The trephine should be 2–3 mm away from the cartilage. Debridement of the necrotic lesion was done through the drill hole. The concentrated BMDC containing mononuclear cells along with 4 mg rhBMP-2 (Hangzhou Jiuyuan, China) was injected into the femoral head using a small trephine with an inner diameter of 1.5 mm and an outer diameter of 3.5 mm. Bone wax was used to block the outlet of the channel and prevent leakage (Figs 5 and 6).

#### Postoperative management and assessment of results

The patients were allowed weight bearing with crutches for 14 days after operation and were allowed full weight bearing without crutches thereafter. Non-narcotic analgesics such as celecoxib were prescribed for pain. They were followed up at the outpatient department at 3, 6, 12 months, and every year thereafter. The clinical follow-up consisted of the determination of preoperative and postoperative serial visual analogue scale (VAS) and Harris hip scores (HHS). Serial AP and frog-leg lateral radiographs were used for the radiographic follow-up. All radiographs were analyzed by three of the authors on a consensus basis. On the serial postoperative radiographs, consolidation of the impacted graft and collapse or progression of collapse and osteoarthritis (OA) were determined. A clinical failure was defined as a Harris hip score below 80 points or if the patients needed further surgical procedure such as total hip arthroplasty or osteotomy for any reason. A radiographic failure was defined as the onset or the progression of collapse or progressive OA according to follow-up radiographs.

### Statistical analysis

All results were expressed as mean ± standard deviation (SD). All data analyses were performed using SPSS version 16.0.0 software (SPSS; IL, USA). Pain and Harris hip scores before and after the CD surgery were compared using paired *t* tests. The overall clinical outcomes were compared statistically using a chi-square test for statistical significance with a 95% confidence interval (*P* < 0.05).

## Additional Information

**How to cite this article**: Gao, F. *et al*. Combined with Bone Marrow-Derived Cells and rhBMP-2 for Osteonecrosis after Femoral Neck Fractures in Children and Adolescents: A case series. *Sci. Rep*. **6**, 30730; doi: 10.1038/srep30730 (2016).

## Figures and Tables

**Table 1:** Patient demographic characteristics in ONFH after femoral neck fractures.

Demographics	Values
Gender (male:female)	38:13
Age (year)	16.3 ± 3.8 (11.4–18.1)
BMI (kg/m^2^)	22.7 ± 3.5
Femoral neck fractures (*n*)	
Sides (left:right)	34:17
Delbet type (I:II:III)	5:31:15
Displaced (*n*, %)	49, 96%
Therapeutic method (Spica: Internal fixation+Spica)	2:49
ONFH-the Association Research Circulation Osseous (ARCO) stage lesions (hips)	
Stage I	3
Stage II	21
Stage III	27
ONFH- China–Japan Friendship Hospital (CJFH) classification (hips)	
Type M	0
Type C	4
Type L1	19
Type L2	11
Type L3	17
Duration of hip symptoms (month)	5.2 ± 7.1
Relationship between diagnosis of osteonecrosis and internal fixation removal	
ONFH without internal fixation removal (*n*)	18
ONFH after internal fixation removal (*n*)	31
Interval from internal fixation removal (month)	5.9 ± 3.8
Interval from femoral neck fractures (month)	16.4 ± 9.2
Length of follow- up (year)	6.8 ± 1.5

*Note*: BMI, Body mass index; ONFH, osteonecrosis of the femoral head.

**Table 2:** Clinical characteristics of the affected hips of all ONFH patients in LPFH and non-LPFH groups in this study.

	LPFH group	non-LPFH group
*n* (hips)	23	28
Duration of hip symptoms (month)	6.1 ± 5.3	5.0 ± 3.9
CJFH classification (hips)	M, 0; C, 4; L1, 19	L2, 11; L3, 17
ARCO stage lesions (hips)		
Stage I	3	0
Stage II	14	7
Stage III	6	21
Medical history: Femoral neck fractures (*n*)		
Delbet type (I:II:III)	3:14:6	2:17:9
Anesthesia method (ESA:GA)	15:8	22:6
Operation time (min)	41.2 ± 6.4	38.8 ± 8.3
Intraoperative blood loss (mL)^*^	31.7 ± 9.1	35.6 ± 7.8
Length of follow-up (year)	6.4 ± 1.8	6.9 ± 2.3

*Note:* CJFH, China–Japan Friendship Hospital; ARCO, Association Research Circulation Osseous; ESA, epidural spinal anesthesia; GA, general anesthesia. *Without bone marrow aspirated from the anterior iliac crest.

**Table 3:** Clinical outcome of the affected hips of all ONFH patients based on the ARCO stage in this study.

ARCO stage	*n*	VAS			HHS			Clinical outcome^#^		
		Pre-Op	FU^*^	*P*	Pre-Op	FU^*^	*P*	Improved	Unchanged	Worsened
Stage I	3	2.7 ± 0.6	1.3 ± 0.6	0.047	88.7 ± 2.1	95.0 ± 3.0	0.046	3	0	0
Stage IIa	5	3.6 ± 1.1	1.8 ± 0.8	0.024	77.8 ± 2.4	90.4 ± 3.1	0.000	5	0	0
Stage IIb	6	5.0 ± 1.8	1.5 ± 0.8	0.003	75.7 ± 2.8	87.2 ± 2.2	0.000	6	0	0
Stage IIc	10	4.6 ± 1.9	1.9 ± 1.0	0.001	74.6 ± 3.0	85.4 ± 3.5	0.000	9	1	0
Stage IIIa	18	6.2 ± 1.5	1.9 ± 0.9	0.000	67.7 ± 6.7	81.9 ± 5.4	0.000	15	3	0
Stage IIIb	6	6.5 ± 1.0	2.0 ± 0.9	0.000	67.8 ± 5.0	85.3 ± 4.3	0.000	5	1	0
Stage IIIc	3	8.0 ± 1.0	2.7 ± 0.6	0.003	57.6 ± 4.2	83.0 ± 5.3	0.003	1	1	1
Total	51	5.4 ± 1.9	1.9 ± 0.9	0.000	71.7 ± 8.1	85.3 ± 5.5	0.000	44	6	1

*Note*: ARCO, Association Research Circulation Osseous.; VAS, visual analogue scale; HHS, Harris hip score. Pre-Op, preoperation; FU, follow-up; *The last follow-up time. ^#^Clinical outcome [26]: ‘Improved’ was defined when significant improvements in pain and function of the affected hip were noted after treatment. ‘Unchanged’ was defined when very little or no changes were noted after treatment. ‘Worsened’ was defined when more pain and less function were noted after treatment.

**Table 4:** Clinical outcome before and after surgical treatment in LPFH and non-LPFH groups.

	Before CD surgery	After CD surgery	*P* value (II)
VAS			
LPFH group (*n* = 23)	4.6 ± 1.8	1.6 ± 0.8	0.000
non-LPFH group (*n* = 28)	6.1 ± 1.7	2.1 ± 0.9	0.000
*P* value (I)	0.003	0.055	
HHS			
LPFH group (*n* = 23)	77.4 ± 5.6	87.5 ± 5.2	0.000
non-LPFH group (*n* = 28)	67.0 ± 6.7	83.5 ± 5.1	0.000
*P* value (I)	0.000	0.008	
	**LPFH group (*****n***** = 23)**	**non-LPFH group (*****n *****= 28)**	***P*** **value (III)**
Clinical outcome^*^			
Improved	91.3% (21/23)	82.1% (23/28)	0.436
Unchanged	8.7% (2/23)	14.3% (4/28)	0.678
Worsened	0% (0/23)	3.6% (1/28)	1.000

*Note*: CD, Core decompression; VAS, visual analogue scale; HHS, Harris hip score.

*P* value (I): comparison of data between LPFH group and non-LPFH group for pain score and Harris hip score.

*P* value (II): comparison of data before and after CD within the same group.

*P* value (III): comparison of data between LPFH group and non-LPFH group for clinical outcome.

*Clinical outcome [26]: ‘Improved’ was defined when significant improvements in pain and function of the affected hip were noted after treatment. ‘Unchanged’ was defined when very little or no changes were noted after treatment. ‘Worsened’ was defined when more pain and less function were noted after treatment.
